# The Majority of Primate-Specific Regulatory Sequences Are Derived from Transposable Elements

**DOI:** 10.1371/journal.pgen.1003504

**Published:** 2013-05-09

**Authors:** Pierre-Étienne Jacques, Justin Jeyakani, Guillaume Bourque

**Affiliations:** 1 Computational and Systems Biology, Genome Institute of Singapore, Singapore, Singapore; 2 Département de Biologie, Université de Sherbrooke, Sherbrooke, Québec, Canada; 3 Department of Human Genetics, McGill University, Montréal, Québec, Canada; 4 McGill University and Génome Québec Innovation Center, Montréal, Québec, Canada; University of Utah School of Medicine, United States of America

## Abstract

Although emerging evidence suggests that transposable elements (TEs) have contributed novel regulatory elements to the human genome, their global impact on transcriptional networks remains largely uncharacterized. Here we show that TEs have contributed to the human genome nearly half of its active elements. Using DNase I hypersensitivity data sets from ENCODE in normal, embryonic, and cancer cells, we found that 44% of open chromatin regions were in TEs and that this proportion reached 63% for primate-specific regions. We also showed that distinct subfamilies of endogenous retroviruses (ERVs) contributed significantly more accessible regions than expected by chance, with up to 80% of their instances in open chromatin. Based on these results, we further characterized 2,150 TE subfamily–transcription factor pairs that were bound *in vivo* or enriched for specific binding motifs, and observed that TEs contributing to open chromatin had higher levels of sequence conservation. We also showed that thousands of ERV–derived sequences were activated in a cell type–specific manner, especially in embryonic and cancer cells, and we demonstrated that this activity was associated with cell type–specific expression of neighboring genes. Taken together, these results demonstrate that TEs, and in particular ERVs, have contributed hundreds of thousands of novel regulatory elements to the primate lineage and reshaped the human transcriptional landscape.

## Author Summary

Nearly half of the human genome is composed of repetitive sequences, most of which were derived from transposable elements that have replicated in the genome during the evolution of our species. There is growing evidence showing that some of these transposon-derived sequences have been a source of new binding sites for various mammalian transcription factors. Considering that previous studies were targeting only few transcription factors and cell types, a key question that remains is to what extent the transposable elements have contributed to human transcriptional networks. To systematically survey this contribution, we used datasets generated by the international Encyclopedia of DNA Elements (ENCODE) consortium, identifying the location of active regulatory elements in more than 40 distinct human cell types. Using this resource we measured the contribution of all classes of repetitive sequences and systematically characterized the impact that transposable elements have had on the human chromatin landscape. Our results demonstrate that transposon-derived sequences have contributed hundreds of thousands of novel regulatory elements to the primate lineage and reshaped the human transcriptional landscape.

## Introduction

Nearly half of human DNA is derived from sequences known as TEs that have successfully replicated in the genome during the evolution of our species [1]. Although the parasitic behavior of TEs was initially put forward as a sufficient explanation for their maintenance within genomes [2], [3] there is growing evidence to support the alternative view that TEs have facilitated genomic innovations [4], [5] and contributed critical regulatory elements to their host [6]. Indeed, a number of studies have shown recently that TEs have been the source of binding sites for various mammalian transcription factors (TFs) [7]–[9] and that they have rewired different developmental regulatory networks [10]–[12]. However, given that previous studies were limited either by the number of TFs they surveyed [7]–[10], [13], [14] or by the cell types they explored [10], [11], [15], a key question that remains is to what extent have TEs globally contributed to human transcriptional networks in undifferentiated and differentiated cells. The importance of characterizing the functional role of TEs and other repetitive regions in the human genome is accentuated by the facts that these sequences constitute most of the sequence diversity between mammalian species [16] and are a significant source of human polymorphisms [17] and of somatic mutations in healthy and disease tissues [18]–[20].

To systematically survey the contribution of TEs to human regulatory networks across a range of cell types, we made use of DNase I hypersensitive sites (DHS) data generated at the University of Washington (UW) and Duke as part of ENCODE [21], [22]. The benefit of using these chromatin accessibility maps is that they highlight active DNA sequences [23], [24] independently of a selected set of TFs. Although accessibility does not equate regulatory function, we build upon these data sets to measure the global activity profile of all classes of transposon-derived sequences and systematically characterize the impact that ancient and recent TEs expansions have had on the human chromatin landscape.

## Results

### TEs have contributed a large fraction of accessible regions in human cells

**Figure 1:**
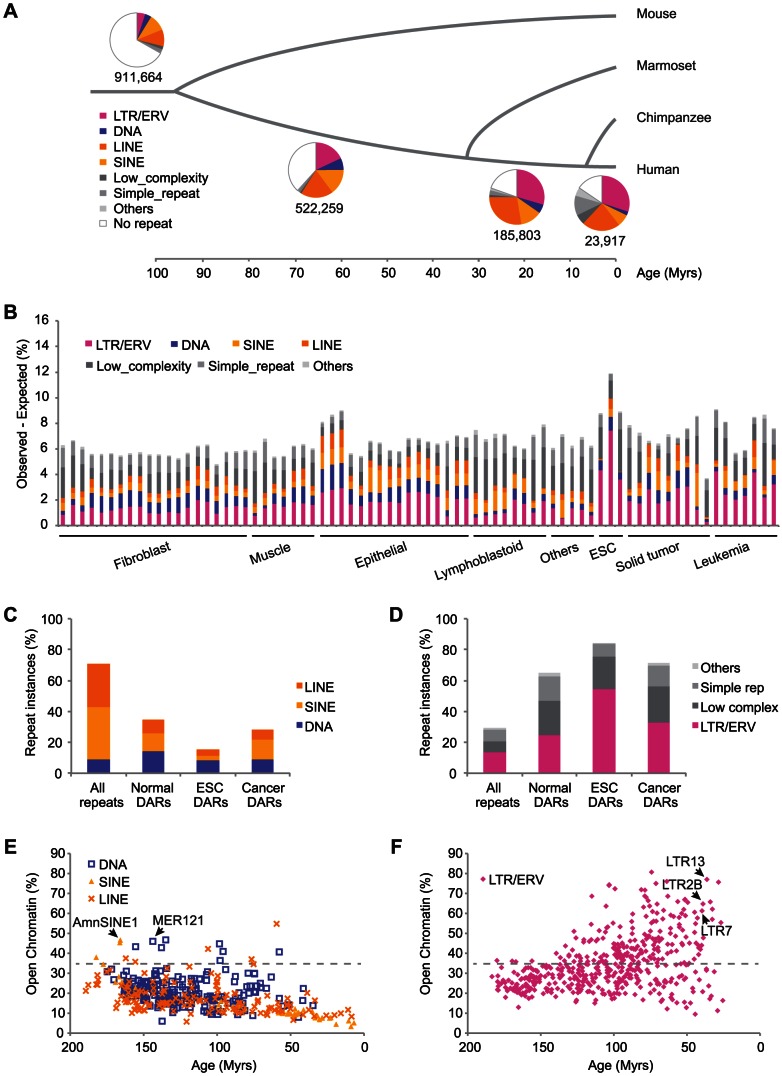
TEs have contributed a large fraction of accessible regions in human cells. (A) Proportion of human DHS regions overlapping different classes of repeats based on the age of the sequence in which they are embedded. (B) Specific repeat subfamilies, called DHS-associated repeats (DARs), are over-represented and their cumulative relative contribution (Observed-Expected) is shown as a percentage of all DHS data. (C–D) Proportion of all repeat instances in the genome (All repeats) and for DAR instances in three classes of cells (Normal, ESC and Cancer). (E–F) Fraction of repeat subfamily instances that is contributing to open chromatin in at least one data set. The estimated age is in millions of years (Myrs).

Starting from 106 DHS data sets we performed extensive quality control and retained 75 data sets defining a total of 11,848,530 regions of open chromatin in 41 distinct human cell types derived from normal, embryonic, and cancer tissues (Table 1 and Table S1, see Materials and Methods). These DHS data were further grouped across cell types into 1,643,643 distinct regions of open chromatin. By measuring the overlap with repeat elements, we found that 725,610 (44.1%) DHS regions overlapped instances of the 4 major classes of TEs (ERV, also known as LTR, DNA, LINE and SINE). Notably, by partitioning the DHS regions based on the presence or absence of homologous sequences at orthologous loci in other species, we also found that this proportion reached 63.1% for elements embedded in primate-specific sequences (Figure 1A, see Materials and Methods). A large fraction of these primate-specific DHS regions were observed in repeat subfamilies that were themselves specific to the primate lineage as estimated from the divergence of the repeat instances from their consensus (Figure S1, see Materials and Methods).

**Table 1:** The 75 DNase I data sets used in this study were grouped in 8 tissues.

Tissue	Cell lines
Fibroblast	Normal, Normal_Park., ProgFib, Neonatal, Fetal_lung (AG04450)†, Toe (AG09309)†, Gum (AG09319)†, Gingival (HGF)†, Abdominal (AG10803)†, Lung (NHLF)†, Skin (BJ-T)†, Cardiac (HCF)†
Muscle	Myoblast (HSMM), Myotube (HSMMtube), Myocytes (HCM)†, Skeletal (SKMC)†, Aortic_smooth (AoSMC)
Epithelial	Small_air (SAEC), Esophageal (HEE)†, Choroid_plex (HCPE)†, Retinal (HRPE)†, Ciliary (HNPCE)†, Renal_cortical (HRCE)†, Renal (HRE)†, Prostate (LHSR), Amniotic (HAE)†
Lymphoblastoid	GM12891, GM19238, GM19239, GM19240, GM12865†, GM18507, GM12878
Others	Myometrial, PanIslets, Melanocytes, Epidermal (NHEK), Endothelial (HUVEC)
hESC	H1esc, H7esc, H9esc
Solid_tumor	HepG2, HeLa-S3†, PANC-1, MCF-7††, Medullobastoma, Neuroblastoma
Leukemia	CMK, HL-60, NB4†, K562†, Jurkat

Cell lines marked with a “†” or “††” had two or three biological replicates respectively.

Given that repeats are ubiquitous in the genome, we wanted to compare the proportion of DHS regions observed in TEs relative to what would be expected by chance. Using annotation-matched random distributions we found that specific repeat subfamilies were significantly over-represented in individual DHS data sets (see Materials and Methods). For example, we observed that 1237 of the 2337 (52.9%) LTR7 repeat instances (a subfamily of the LTR/ERV class) were contributing to open chromatin in the human embryonic stem cell (ESC) line H7 when we would have only expected 60.5 (2.6%). This corresponds to a 20-fold enrichment and is highly significant (*p*<1.0E-100). We call such repeat subfamilies DHS-associated repeats (DARs) and, using a stringent cutoff (*p*<1.0E-05), we identified 8937 DARs enriched in various cell types (Table S2). These DARs provided on average 6.7% and up to 11.9% more open chromatin regions than expected by chance in the data sets surveyed (Figure 1B).

We were interested in characterizing further the families of TEs that were contributing to regions of open chromatin. By combining the DAR instances across the various cell types and comparing them to the number of repeat instances of each family across the genome, we found that LINE and SINE repeats were depleted while DNA repeats were observed at levels expected (Figure 1C). In contrast, we found that LTR/ERV, Low complexity, Simple repeats and Others repeat classes were enriched (Figure 1D). For example, although LTR/ERV repeats constitute 13.5% of the repeat instances in the genome, they represent 25.0%, 54.6%, and 33.0% of the DAR instances in normal, embryonic, and cancer cells, respectively. The over-representation of LTR/ERVs in DHS corroborates an observation made previously [22] and did not appear to be a consequence of intrinsic properties of the repeat subfamilies including mappability (Figures S2 and S3, see Materials and Methods). Low complexity, Simple repeats and Others repeat classes were excluded from most downstream analyses because of their extreme GC content (Figure S3) potentially affected by sequencing biases [25].

Next, we looked at the fraction of instances in all repeat subfamilies that were contributing to open chromatin in relation to their estimated age (see Materials and Methods). We observed that for SINE, LINE, and DNA repeats, older subfamilies tended to contribute more often to open chromatin (Figure 1E). Two of the subfamilies contributing the most were AmnSINE1 and MER121, both previously suggested to have acquired functionality in the host [26], [27]. Intriguingly, we observed the reverse pattern for the LTR/ERV repeats with many of the young subfamilies contributing to open chromatin at very high levels (e.g. LTR13 with 379 instances contributing to open chromatin out of 492 (77.0%), LTR2B with 215 out of 332 (64.8%), and LTR7 with 1432 out of 2337 (61.3%)) (Figure 1F). This pattern although dampened was also visible if we restricted the analysis to the data sets derived from normal differentiated tissues (Figure S4).

### DARs are enriched for binding motifs, occupied by TFs, and show sequence conservation

**Figure 2:**
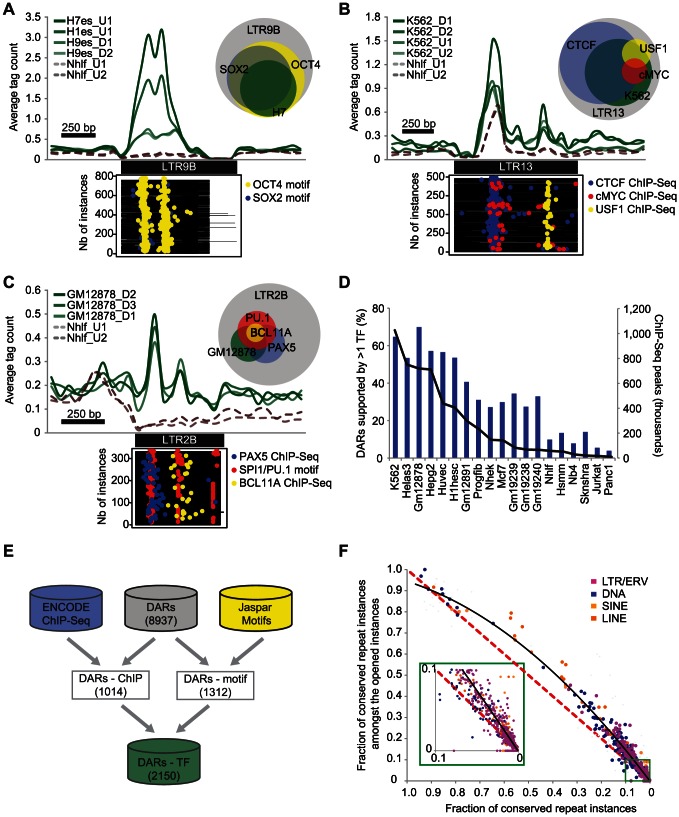
DARs are enriched for binding motifs, occupied by TFs, and show sequence conservation. Aggregate profiles of DNase I tags (green) over the instances of different DARs: (A) LTR9B in ESC, (B) LTR13 in K562 and (C) LTR2B in GM12878. The profiles over another cell type (Nhlf) are shown as a control (dashed brown lines). The plots underneath the profiles represent the localization of regulatory motifs or ChIP-Seq peaks in the same cell lines (yellow, blue, red points). The Venn diagrams represent the proportion of repeat instances (grey) containing DHS and regulatory motifs or ChIP-Seq peaks using the same color code. (D) Proportion of DARs with at least one enriched TF (blue bars) and the total number of binding sites reported (black line) for each cell line. (E) Diagram showing the number of DARs supported by at least one TF based on ChIP-Seq or motif enrichment. (F) Scatterplot showing for each repeat subfamily, the fraction of conserved repeat instances amongst the opened instances. The black line represents a polynomial trend line of order 2. The red dashed line is the expected distribution.

We noted that DHS overlapping repeats were enriched in chromatin states corresponding to promoters, enhancers, and insulators as defined previously using histone marks profiles [28] (Figure S5). To understand why specific TEs were contributing to open chromatin, we wanted to integrate the DARs with other more targeted functional genomics data sets. For example, it was shown previously that the pluripotency TF OCT4 was bound on LTR9B repeats [10] and it was interesting to see the same repeat subfamily as a DAR in ESCs (Table S2). When we looked in LTR9B for the binding motifs of OCT4 and SOX2, another pluripotency TF, we found them to be specifically over-represented in the repeat instances contributing to open chromatin (*p* = 6.8E-64 and 3.6E-38 respectively, see Materials and Methods). Notably, the peaks in the aggregate read density profiles of the DHS data in ESCs were also correlating with the localization of the motifs within the repeat instances (Figure 2A).

To characterize more systematically the role of repeat instances in the host genome and to identify putative functional factors associated with the DARs, we used a collection of TF binding sites determined by ENCODE using ChIP-Seq. In the 19 cell types where both DHS and ChIP-Seq data were available, we found that 1014 of the 2784 DARs (36.4%) were statistically enriched for at least one TF (Table S3). This relied on two statistical tests: one that showed that the TF was enriched in the same repeat subfamily and in the same cell type, and one that showed that the number of instances with both DHS and ChIP-Seq signal was also significant (see Materials and Methods). Using this strategy, we found for example that 82.9% of the 210 LTR13 instances that were contributing to open chromatin in K562 were also bound by CTCF (*p* = 1.1E-13, Figure 2B). Additional DARs supported by specific TFs such as PU.1, BCL11A, and PAX5 in LTR2B are shown in Figure 2C and Figure S6. Predictably, we found that a larger fraction of DARs can be explained by the binding of specific TFs in cell lines where more ChIP-Seq data sets were available (Figure 2D).

To improve on the limited ChIP-Seq coverage in some cell types and in order to characterize the DARs more comprehensively, we developed a classifier to predict TF-repeat associations using Jaspar TF binding motifs (see Materials and Methods). Using this classifier we were able to suggest 3073 high-confidence motif-repeat subfamily associations for 1312 DARs (Table S4). By combining both methods, we were able to predict a total of 2150 unique TF-repeat subfamily associations, which suggest potential functional candidates for 24.1% of the DARs (Figure 2E).

Finally, to further confirm the functional importance of DARs, we also used the annotated conserved non-exonic elements (CNEEs) [29] and assessed the overall sequence conservation of the TEs that were contributing to open chromatin. In total, while only 5.5% of all repeat instances were conserved, we found that 9.0% of the repeats contributing to open chromatin were conserved, a difference that is highly significant (*p*<1.0E-100, Figure S7). Notably, for almost all repeat subfamilies, we found that the subset of instances contributing to open chromatin was more conserved than expected by chance (Figure 2F).

### Thousands of LTR/ERV sequences are activated in a cell type–specific manner especially in ESCs

**Figure 3:**
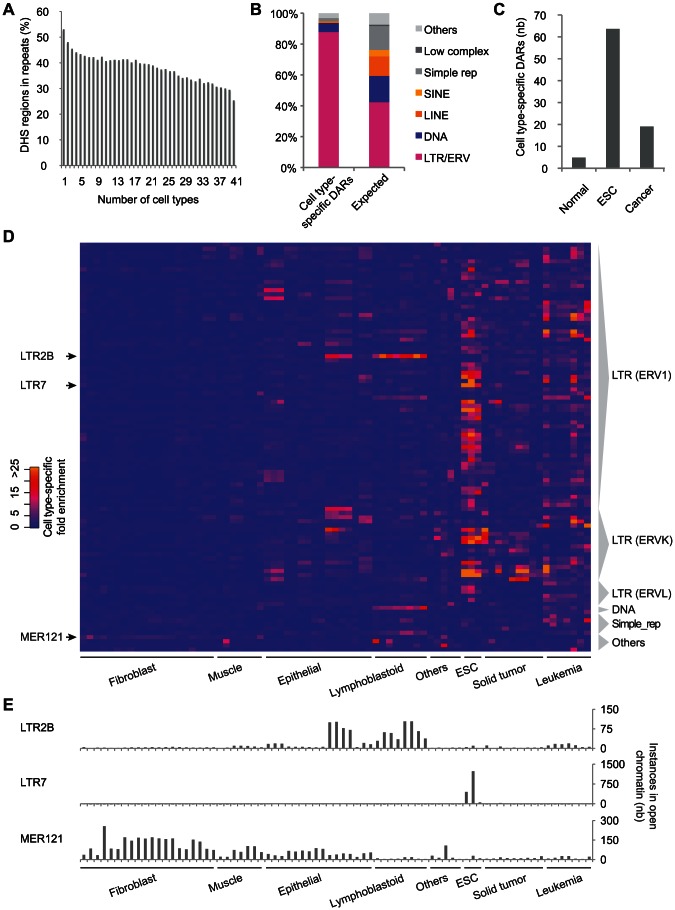
Thousands of LTR/ERV–derived sequences are activated in a cell type–specific manner, especially in ESCs. (A) Proportion of DHS clusters that overlap repeats based on the number of distinct cell types in which they are observed. (B) Proportion of cell type-specific DARs and of all repeat subfamilies (Expected) by repeat class. (C) Average number of cell type-specific DARs per data set in normal, embryonic and cancer cell lines. (D) Heatmap showing the cell type-specific fold enrichment for the top 100 repeat subfamilies. (E) Number of instances contributing to open chromatin for the LTR2B, LTR7 and MER121 repeat subfamilies.

Next, we were interested in the contribution of repeats to cell type-specific DHS. When we calculated the number of cell types contributing to individual DHS regions, we found that 76.0% of the loci were open in 4 cell types or less (Figure S8, see Materials and Methods). We also observed that regions contributed by few cell types were found more frequently in repetitive sequences (Figure 3A). To determine the cell type-specificity of each repeat subfamily we used the median number of open instances in all DHS data sets as the denominator and calculated the fold enrichment for each repeat subfamily in each cell type (see Materials and Methods). A total of 770 DARs showed a cell type-specific fold enrichment greater than 3 (Figure S8 and Table S2). Notably, we observed that LTR/ERV repeat subfamilies were over-represented in the cell type-specific DARs (Figure 3B) and that on average a higher number of cell type-specific DARs were found in ESCs and cancer data sets (Figure 3C). These patterns were also recapitulated in the top 100 repeat subfamilies with the greatest cell type-specific enrichment (Figure 3D). For example, in the case of the LTR7 repeat subfamily, we observed a remarkable enrichment of 131.6- and 88.7-fold in the ESC lines H7 and H1 respectively. While most cell type-specific DARs were found in ESCs and cancer cell lines, we also found examples, such as the LTR2B and MER121 subfamilies, which had most of their instances in open chromatin from normal differentiated cells (Figure 3E). Additional examples of cell type-specific DARs are shown in Figure S9. We also found that the cell type-specificity of various subfamilies of TEs was supported by the chromatin states previously described [28]. For example, more than 40% of the LTR2B instances were annotated as enhancers in GM12878 while only 10% were annotated as such in H1. In contrast, more than 40% of the LTR7 instances were annotated as enhancers in H1 while only 2.2% of them were annotated as such in GM12878 (Figure S10).

Finally, using a collection of TF binding motifs including novel motifs identified in DNase I footprints [30], we identified tissue-specific motifs enriched in these cell type-specific DARs (Table S5, see Materials and Methods). In particular, we observed that many ESC-specific DARs were supported by ESC-specific motifs that were not enriched in normal- or cancer-specific DARs (Figure S11). The top three ESC-specific motifs found in this way were OCT4, SOX2 and KLF4.

### DARs are associated with cell type–specific expression and over-represented in dsQTLs

**Figure 4:**
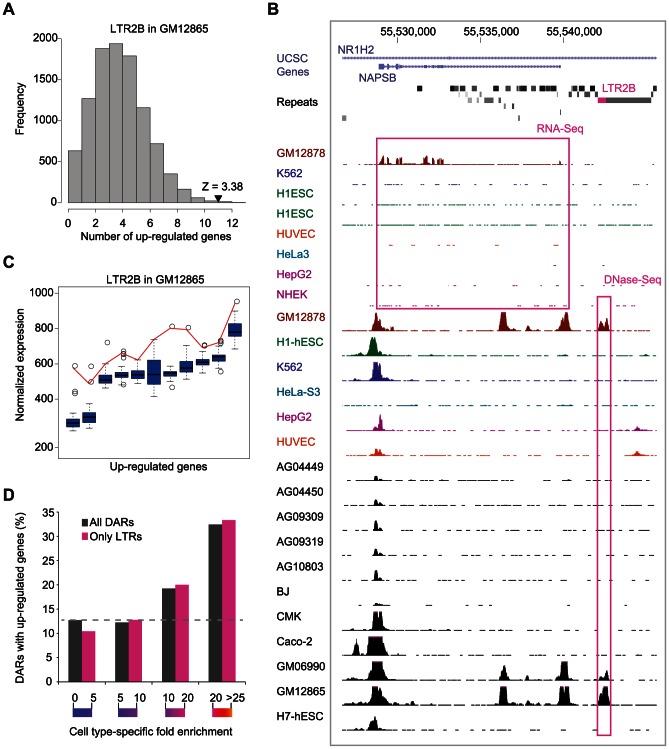
Cell type–specific expression of DAR–associated genes. (A) Distribution of the expected number of up-regulated genes in proximity to the LTR2B DAR instances in GM18265. Actual number of up-regulated genes is shown using an arrowhead. (B) UCSC genome browser view of the NAPSB gene with selected RNA-Seq and DHS ENCODE tracks (y-axis maximum set to 20 and 100 respectively). The LTR2B repeat is highlighted in pink along with its cell type-specific open chromatin and expression profiles. (C) Boxplots showing the expression values across cell types for the DAR-associated genes that are up-regulated. Red lines are connecting the expression values observed in GM18265. (D) Cell type-specific DARs have more cell type-specific expression. DARs were binned according to their cell type-specific fold enrichment and the proportion of them having a *Z-score* of cell type-specificity expression above 3 is shown.

To evaluate the impact of DARs on gene regulation, we used 43 gene expression exon-array data sets from ENCODE and calculated the number of genes in proximity to DAR instances that were up-regulated in the relevant cell type relative to the others (see Materials and Methods). We identified 783 DARs with more proximal up-regulated genes than expected by chance (Table S6). For example, we identified 11 genes in proximity to LTR2B instances that were up-regulated in GM12865 while we would have only expected 4.27 (Figure 4A). Examples of cell type-specific LTR2B associated genes in GM12865 include NAPSB and CLECL1 (Figure 4B and Figures S12, S13), two genes that have been shown to play a role in lymphoblastoid cells [31], [32]. Moreover, we observed that the expression of the DAR-associated genes were frequently highest in the cell type where the DAR had been identified (Figure 4C and Figure S14). We also found that DARs with a higher cell type-specificity score had a higher chance of being associated with cell type-specific expression (Figure 4D). Similar results were obtained using ENCODE RNA-Seq data sets generated by Caltech (Figure S15).

Finally, a recent study combining genotypes with DHS data in 70 lymphoblastoid cell lines has shown that a significant proportion of open chromatin regions, known as dsQTLs, can be influenced by polymorphisms [33]. Having demonstrated that DARs exhibit features associated with regulatory elements, we wanted to test if they also showed this variation across individuals. We found that 36.8% of the reported dsQTLs overlapped repeat instances and that these were contributed by DAR instances in lymphoblastoid cells more than expected by chance (*p* = 1.1E-6, see Materials and Methods).

## Discussion

In summary, we found that TEs have contributed nearly half of the open chromatin regions of the human genome and the majority of primate-specific elements. This estimate is a lower bound that is likely to grow given that better strategies using longer and paired-end reads will be needed to measure the contribution of young repeat subfamilies and polymorphic sites (Figure S3). An example is the L1PA2 repeat subfamily where, despite the fact that the mappability ratio is 0.08, 117 and 257 of the 4904 L1PA2 instances contributed to the H1 and H7 DHSs respectively. This finding is consistent with previous observations [7], [15], [34], [35] but greatly expands on our understanding of the repeat families contributing to open chromatin in the human genome.

To better understand the regulatory functions that could have been retained in exapted TEs beyond the ones that have already been studied (e.g. [8]–[11], [14]), we predicted a total of 2150 TF-repeat subfamily associations and confirmed that a broad range of functional proteins are targeting these regions (Figure 2 and Tables S3, S4). This resource will be useful to provide insights into the regulation of some of the TE-derived loci that have already been implicated in disease [36]. There is an important distinction between biochemical activity and functional relevance to the host. To help confirm the importance of these regions, we also showed that repeat instances contributing to open chromatin were more conserved than expected by chance (Figure 2F).

Next, we demonstrated that LTR/ERV repeats have contributed a disproportionate fraction of cell type-specific accessible chromatin regions especially in embryonic and cancer cell lines (Figure 3). This is interesting given that network rewiring using ERV elements has already been described in ESCs [10]–[12] and that it has been shown that stem cell potency fluctuates with endogenous retrovirus activity in mouse [37]. The level of activity observed in ERV sequences is likely a consequence of the permissive chromatin state found in ESCs that it sometimes reinstated in cancer [38]. There is fine balance between the successful replication of endogenous retroviruses, from which these repeats are derived, and retrotransposition control in the host [39]. One intriguing possibility is that the manipulations that were initially exerted by the ancestral viruses on their host to by-pass these control mechanisms have also facilitated co-option [40].

Finally, we also reported that repeat subfamilies activated in a cell type-specific manner were also frequently associated with higher expression of neighboring genes. This result corroborates the fact that at the level of expression, TE-derived transcripts, including lincRNAs [41], are also usually tissue-specific [42]. Interestingly, this pattern was observed not only in ESCs but also in differentiated and cancer cells (Figure 4 and Table S6).

Taken together, these results demonstrate that TEs, and in particular endogenous retroviruses, have considerably transformed the transcriptional landscape during primate evolution.

## Materials and Methods

### DNase I hypersensitive sites datasets

We retrieved 106 ENCODE DHS data sets available from the October 2010 freeze which included replicates for 50 different cell types from a variety of normal differentiated cell types, human ESCs and cancer cell types using the UCSC ENCODE portal (http://genome.ucsc.edu/ENCODE/). These data had been generated from performing DNase I digestion of intact nuclei, isolating DNase I digested fragments and direct sequencing of fragment ends [23], [24]. We discarded 3 data sets involving treatments and performed extensive quality control of the data sets. Specifically, for each peaks file generated by UW corresponds one tagAlign file such that we calculated the average GC content of the tags and removed data sets with GC bias (>55% or <45%). We also used FastQC (http://www.bioinformatics.babraham.ac.uk/projects/fastqc/) on these files to remove data sets that failed the per sequence quality criteria (when the most frequently observed mean quality is below 20) or where the total number of overrepresented sequences was above 1 million. Multiple tagAlign files generated by Duke had been combined to call the peaks and we analyzed the individual tagAlign files as described above. The quality control summary is presented in Table S1. In the end, we retained 75 data sets that did not have any unusual deviation based on our quality metrics: 51 produced by UW and 24 by Duke, covering 41 different cell types grouped in 8 “tissues” (Table 1). Most of the UW data sets had a read length of 36 bp while the Duke data sets had a read length of 20 bp. For the downstream analyses, we used the narrow peak files that were generated from the uniquely mapped reads and provided by ENCODE.

### Overlap between DHS regions and repeats

The peak regions from the 75 data sets were combined and those within less than 100 bp were grouped in 1,643,643 distinct DHS regions. These DHS regions were resized to 200 bp using their middle point before intersecting with the 5,269,366 repeat instances of RepeatMasker [43] from the UCSC Genome Browser [44] (Figure 1A). In order to calculate the proportion of DHS regions overlapping different classes of repeats based on the age of the sequence in which they are embedded, we sequentially used the liftOver utility from UCSC using default parameters and minMatch set to 0.5. The DHS regions (hg18) were first converted to the mouse genome (mm9), those not converted were then mapped to the marmoset genome (CalJac3), and again those not converted were mapped to the chimp genome (PanTro2) to identify the 23,917 human-specific DHS regions. Primate species divergence time taken from [45]. We also obtained an alignment-free estimate of the age of the repeat subfamilies using the average divergence between the instances and the ancestral repeat consensus (milliDiv value reported by RepBase) and applying the Jukes Cantor method with a substitution rate of 2.2×10-9 per site per year [7]. The ages obtained using this method were largely consistent with previous estimates [16], [46]. Repeat subfamilies with an estimated age <95 Myrs were said to be primate-specific.

### Annotation-matched random distributions and DAR identification

For each DHS data set, we calculated the number of overlaps within each repeat subfamily using a 200­bp window surrounding the center of the DHS peaks. Next, following a strategy developed on ChIP-Seq data sets [7], [10], we annotated each DHS with respect to its nearest RefSeq genes and we binned the DHS into six categories according to the peak location: TSS (within 1 kbp of a TSS), promoter (up to 5 kbp upstream of TSS), intragenic (within the RefSeq gene boundary), proximal (up to 10 kbp away from the gene boundaries), distal (up to 100 kbp away from the gene boundaries) and desert (more than 100 kbp away from any RefSeq genes). We then generated for each DHS data set a random set of 200,000 regions with the same annotation distribution as the true regions and intersected with the RepeatMasker track to obtain the expected number of overlaps for each repeat subfamily. We used a one­sided binomial test to compare the observed number of repeats intersecting the true DHS with the expected numbers from the annotation­matched background. We identified repeat subfamilies with statistically significant contribution to open chromatin (*p*<1E-5) as DHS-associated repeats (DARs).

### Properties of repeat subfamilies and mappability ratio

We verified that various properties of the repeat subfamilies were comparable across repeat classes. Specifically, we checked for an association between the fraction of repeat instance contributing to open chromatin and the number of instances in a given subfamily, average size and GC content (Figure S2). We only detected an association between open chromatin contribution and GC content affecting Low complexity, Simple repeats and Others repeat classes, which we excluded from most analyses. The majority of the ENCODE processed data sets, such as the narrow peak files that we used in the current study, rely on uniquely mapped reads [47], [48]. To test the impact of such a criteria on the detection of TEs regulatory activity, we extracted 50 million 20 bp and 36 bp sequences (to mimic Duke and UW read lengths respectively) from random location on the human and re-mapped these artificial reads using the Bowtie program [49] and allowing 1 and 2 mismatches respectively. The goal of these simulations was to compute a mappability ratio for each repeat subfamily that is: the ratio between the number of uniquely mapped tags in a particular subfamily and the number of tags that were extracted from this subfamily (Figure S3).

### Chromatin states analyses

The DHS from the six cell lines (some with two replicates for a total of eight DHS data sets) for which the chromatin states (CS) were available [28] were used for this analysis. The DHS were associated to one of the 15 CS (overlap >50%) using intersectBed from BEDtools [50], then grouped whether they overlap any repeat instance or not. For each data set, the proportion of DHS annotated as each CS was computed, and similar CS were combined together (ex: strong and weak enhancers grouped as enhancers). Similarly, random DHS (using shuffleBed) were generated and overlapped with the CS. Figure S5 is showing the average and standard deviation over the eight data sets. Repeat instances from specific DARs (LTR7, LTR2B and LTR13) were intersected with the CS using a similar strategy (Figure S10).

### ChIP–Seq analysis

We used 183 distinct ENCODE ChIP-Seq data sets generated by the Broad Institute, Duke, HudsonAlpha and Yale, covering 87 different TFs from 19 cell lines for which we also used DHS. For each of these data sets we applied the same procedure as to identify the DARs and identified a total of 9367 TF-repeat subfamily pairs. These pairs were then intersected with the DARs and for each combination from the same cell type and the same repeat subfamily we applied a hypergeometric to test the significance of the number of instances with ChIP-Seq peak and DHS. Using a stringent cutoff (*p-value*<0.001), we identified 2800 statistically significant combinations of DAR-ChIP Seq for 1014 distinct DARs (Table S3).

### Motif analysis

Using ChIP-Seq data sets obtained previously [51], we trained a classifier that uses the over-representation of TF binding motifs and other features of repeat subfamilies to predict TF-repeat associations. Briefly, five features of repeat subfamilies were used: 1) fraction of repeat instances with motif, 2) fraction of motifs contained within repeat subfamily, 3) motif score ratio between bound and unbound repeat instances, 4) enrichment test for binding motifs within repeat subfamilies, 5) simulations looking at the potential of repeat sequences to generate binding motifs. By combining these individual features using a weighted rank average we were able to achieve an Area-Under-the-Curve (AUC) of 0.81 for this classifier (Jeyakani et al., in preparation). Using 103 JASPAR TF binding motifs derived from human or mouse [52], we applied our classifier to the list of putative motif-repeat subfamily pairs and using a stringent cutoff (top 10%) we identified 2337 potential associations. These motif-repeat subfamily pairs were then intersected with the DARs and for each combination from the same repeat subfamily we applied a hypergeometric to test the significance of the number of instances with motifs and DHS. Using a stringent cutoff (*p-value*<0.001), we identified 3857 statistically significant combinations of DAR-motif from 1312 distinct DARs (Table S4).

### Cell type specificity and motif enrichment

In order to calculate the cell type-specific enrichment for each repeat subfamily, we determined the median number of repeats bound across the DHS data sets (these numbers were further normalized to the total number of sites in each data sets). The median value was computed independently for the UW and the Duke data sets because of the expected differences in mappability given the differences in read lengths. Next, we calculated a cell type-specific fold enrichment for each repeat subfamily in a given cell type by dividing the observed number of repeats contributing to open chromatin in this particular DHS data set by the median number of repeats contributing to open chromatin for this subfamily. This was done for all DARs and non-DARs (Figure S8). Next, we scanned the 56,837 DHS from the 770 cell type-specific DARs for motifs using the FIMO software tool with a maximum p-value threshold of 1×10−5 as was done in [30]. We provided motif templates from Jaspar [52], TransFac [53], Uniprobe [54] and novel de novo motifs identified previously in DNAse I footprints [30]. For each DAR, we then identified the motifs present in >25 repeat instances and in >20% of the instances contributing DHS (Table S5). We also calculated the proportion of DARs from ESC, Cancer and differentiated normal cells with support of at least one of the 28 ESC-specific motif identified in [30] (Figure S11).

### Expression analyses

From the 70 ENCODE UW Affy All-Exon Arrays expression data sets, only those from the cell types with DHS were selected. These data sets were clustered showing that a few replicates were inconsistent and therefore removed of the downstream analyses, leaving 43 expression data sets (most of them in duplicate). Note that we tried to combine these data sets with the ENCODE Duke Affy All-Exon Arrays data sets but found that the platform correlation was higher than the biological correlation between biological replicates so we therefore decided to only use the UW data (data not shown). Expression data was available in the cell type for 6054 of the 8937 DARs. A gene was called up-regulated in a cell type if it had a *Z-score* >2 in at least one of the data set compared to the other data sets. For each DAR, the *Z-score* of cell type-specific expression based on the number of up-regulated genes was computed on permutation tests by randomly picking 10,000 times the same number of genes that associated with the DAR from the set of 35,865 different gene names covered by the arrays. For example, from the 2337 instances of LTR7 in the genome, 788 were contributing to open chromatin in H7 and those were associated to a total of 561 distinct genes. The fact that 85 of these genes were up-regulated in the ESC cell type while only 19.6 (+/−4.3) were expected based the permutation test gives a *Z-score* of 15.1 (Figure S13 and Table S6).

Similarly, 13 RNA-Seq data sets generated by Caltech from 7 distinct cell types were used to calculate the association of DARs with up-regulation of expression. A 50 kb window centered in the middle of each repeat instance was used and, to estimate the background, the genome was independently segmented in non-overlapping 50 kb windows. For each RNA-Seq data set, the average tag density was calculated in each window. For each window, the mean and SD of the average tag density was then calculated across the 13 RNA-Seq data sets in order to identify up-regulated windows defined as a *Z-score* >2 in one of the data set of the same cell type compared to the other data sets. For the 2124 DARs for which expression data was available, the *Z-score* of cell type-specificity expression was computed using permutation tests by randomly picking 10,000 times the same number of windows than the number or repeat instances contributing to open chromatin in this cell type from the background genomic segments (Figure S14).

### dsQTLs analysis

The 1,034,427 DHS from the 8 lymphoblastoid DHS data sets were first grouped into 430,159 clusters as described above. Using intersectBed, we found as expected that most (4891 of 6070 (80.6%)) short dsQTLs from [33] were overlapping these DHS lymphoblastoid clusters. We also found that 2234 of 6070 (36.8%) short dsQTLs were overlapping a repeat instance. Considering that 995 of the 4891 (20.3%) short dsQTLs overlap one of the 77,135 DHS lymphoblastoid clusters contributed by DAR instances in lymphoblastoid cells (17.9% of all lymphoblastoid clusters), this overlap is highly significant (hypergeometric *p = *1.11E-6). Doing the same for the DHS lymphoblastoid clusters that were overlapping DAR instances from the other cell types gave a more marginal enrichment (hypergeometric *p = *1.09E-2).

## Supporting Information

Figure S1 Proportion of DHS overlapping primate-specific repeats in each branch of Figure 1A. Repeat subfamilies were defined as primate-specific based on the average divergence of their instances relative to their respective repeat consensus (see Materials and Methods).
(PDF)

Figure S2 For each repeat subfamily, proportion of instances contributing to open chromatin in at least one data set (y-axis) relative to: (A) the number of instances, (B) their average size and (C) their average GC content. The only strong correlation is observed between the proportion of instances of Low_complex, Simple_rep and Others repeat subfamilies in open chromatin and GC content.
(PDF)

Figure S3 (A) Coverage of uniquely mapped short reads on repetitive regions. Fifty million random locations were selected from the human genome. For each of these locations 20 bp and 36 bp sequences were extracted to mimic UW and Duke DHS data sets. These artificial reads were re-mapped using Bowtie allowing for 1 mismatch and 2 mismatches respectively. For each repeat subfamily, a mappability ratio was computed as the number of reads uniquely mapped to this family divided by the number of artificial reads coming from this family. Overall we found that 36 bp reads perform significantly better than the 20 bp reads and 75% of the repeat families have a mappability ratio above 0.8. (B–C) Proportion of simulated reads that can be unambiguously mapped to the reference genome for all repeat subfamilies organized by class. Estimated age in millions of years (Myrs).
(PDF)

Figure S4 (A) Fraction of repeat instances in each DNA, SINE and LINE subfamily that is contributing to open chromatin in at least one normal data set. (B) Same for repeat subfamilies from the LTR/ERV class. In contrast to Figure 1E–1F this analysis is restricted to the normal cell lines.
(PDF)

Figure S5 DHS overlapping repeats are enriched in active chromatin states at a similar level than the DHS outside repeats, and are enriched compared to a random distribution of the DHS. The 15 original states were combined into seven distinct states. The averages and standard deviations are calculated over the eight cell types for which DARs were identified, on the proportion of DHS overlapping (blue) or not (red) repeats or over random distribution (green).
(PDF)

Figure S6 Complement of Figure 2 showing additional TF-repeat associations. Aggregate profiles of DNaseI tags (green) over the instances of different DARs: (A) LTR22 in HEPG2, (B) LTR15 in K562, (C) LTR41 in K562 and (D) LTR21B in K562. The profiles over another cell type (Nhlf) are shown as a control (dashed brown lines). The point's plots underneath the profiles represent the localization of regulatory motif or ChIP-Seq peaks in the same cell lines (yellow, blue, red points). The Venn diagrams represent the proportion of repeat instances (grey) containing DHS and regulatory motifs or ChIP-Seq peaks using the same color code.
(PDF)

Figure S7 Repeat instances contributing to open chromatin tend to be more conserved than expected. Venn diagram showing the overall overlap of 87,219 between the Repeatmasker instances [43], the annotated conserved non-exonic elements (CNEEs) [29] and the DHS (this study).
(PDF)

Figure S8 (A) Distribution of the number of cell types for all DHS regions showing that 75% of the clusters were contributing to open chromatin in 4 cell types or less. (B) Proportion of DARs and non-DARs repeat subfamily by bin of cell type-specific fold enrichment computed for each repeat subfamily in each data set.
(PDF)

Figure S9 Cell type specific DAR examples. (A–C) LTR2B, LTR7 and MER121 from Figure 3. (D) LTR13 which is bound by CTCF (Figure 2B) and contributing to open chromatin in almost all cell type. (E) LTR1 which is showing re-activation of repeats in ESCs and some cancer cell lines but not in the others. (F) LTR47B which is contributing to open chromatin in many cell types except the lymphoblastoids, ESCs and leukemias. (G) LTR10C which is specifically contributing to open chromatin in HEE and SAEC epithelial cell types. (H) LTR10A which is contributing to open chromatin in few epithelial cell types as well as in solid tumors. (I) LTR72 which is contributing to open chromatin in many normal cell types and leukemia.
(PDF)

Figure S10 Cell type-specific DARs are also supported by chromatin-state data. Repeat instances from specific DARs were intersected with the CS in the cell type where the DAR was identified (LTR7 in H1 and LTR2B in GM12878) but also in the other cell type. LTR13 was observed as a DAR in both H1 and GM12878. The CS result for LTR13 is consistent with the fact that the insulator protein CTCF was also enriched in this repeat subfamily (Figure 2B).
(PDF)

Figure S11 (A) Cell type-specific DARs with an ESC-specific motif as defined in [30] in different fraction of the DAR DHS instances (>.2, >.25, etc.) and with at least 25 motif instances. (B) ESC-specific motifs that are enriched in the ESC-specific DARs (with at least 25 motifs and >.2 of the DAR DHS instances). Only the most abundant motif per DAR is shown, but all combinations are available in Table S5.
(PDF)

Figure S12 UCSC genome browser view of the NAPSB gene with RNA-Seq and DHS ENCODE tracks. The LTR2B repeat is highlighted in pink along with its cell type-specific contribution to open chromatin and expression profiles.
(PDF)

Figure S13 UCSC genome browser view of the CLECL1 gene with RNA-Seq and DHS ENCODE tracks. The LTR2B repeat is highlighted in pink along with its cell type-specific contribution to open chromatin and expression profiles.
(PDF)

Figure S14 Cell type-specific expression of DAR-associated genes. (A) Distribution of the expected number of up-regulated genes in proximity to the DAR instances for LTR7 in H7 (left) and LTR15 in K562 (right). Actual number of up-regulated genes is shown using an arrowhead together with the corresponding *Z-score*. (B) Boxplots showing the expression values across cell types for the DAR-associated genes that are up-regulated. Red lines are connecting the expression values observed in the relevant cells.
(PDF)

Figure S15 Cell type specific expression of the DARs based on RNA-Seq data. (A) Distribution of the expected number of up-regulated genes in proximity to the DAR instances for LTR7 in H7 (left), LTR15 in K562 (middle), and LTR2B in GM18265 (right). Actual number of up-regulated genes is shown using an arrowhead together with the corresponding *Z-score*. (B) Boxplots showing the expression values across cell types for the DAR-associated genes that are up-regulated. Red lines are connecting the expression values observed in the relevant cells. (C) Cell type-specific DARs tend to have more cell type-specific expression. DARs were binned according to their cell type-specific fold enrichment and the proportion of them having a *Z-score* of cell type-specificity expression above 3 is shown.
(PDF)

Table S1 List of the 106 ENCODE DHS data sets. The ordering of the 75 retained dataset is included, as well as the quality control analysis for the 31 data sets that were removed.
(XLS)

Table S2 List of the 8937 DARs along with their proprieties and summary of their TF ChIP-Seq and motifs support as well as number of up-regulated genes and corresponding *Z-score*.
(XLS)

Table S3 DARs enriched for TFs ChIP-Seq and their statistics.
(XLS)

Table S4 DARs enriched for TFs motifs and their statistics.
(XLS)

Table S5 Motifs enriched in cell type-specific DARs.
(XLS)

Table S6 DARs enriched for up-regulated genes from exon-array.
(XLS)
