# Supplementary figures and images for: The Majority of Primate-Specific Regulatory Sequences Are Derived from Transposable Elements
Source: PLoS Genet. 2013 May 9;9(5):e1003504. doi: 10.1371/journal.pgen.1003504 (PMC3649963; doi:10.1371/journal.pgen.1003504)

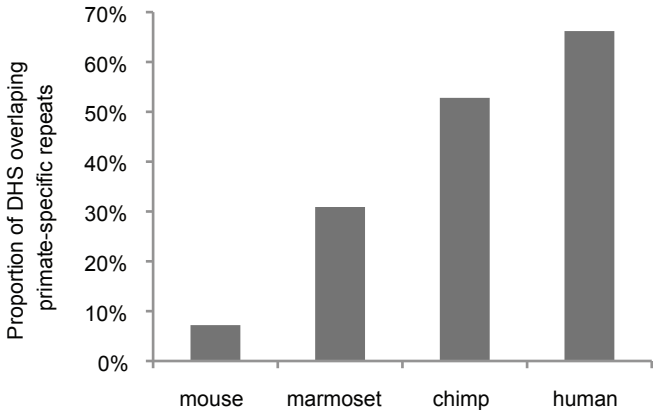

Supplement: Figure S1 — Proportion of DHS overlapping primate-specific repeats in each branch of Figure 1A. Repeat subfamilies were defined as primate-specific based on the average divergence of their instances relative to their respective repeat consensus (see Materials and Methods). (PDF) [file pgen.1003504.s001.pdf]

**A**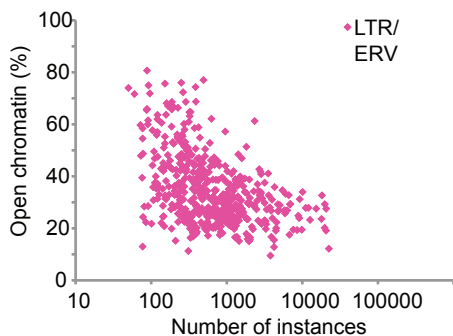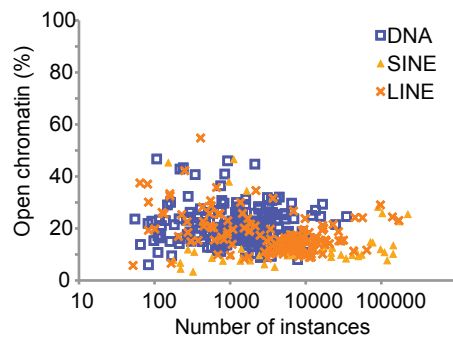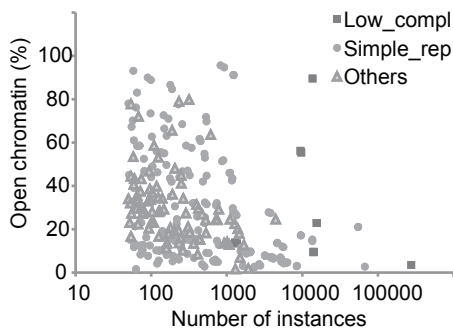**B**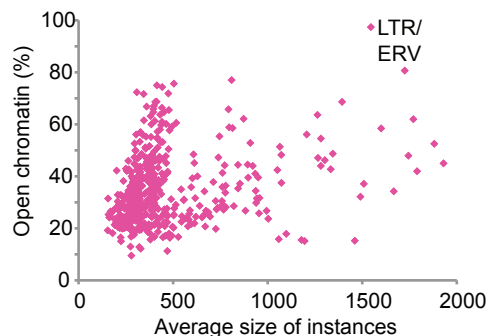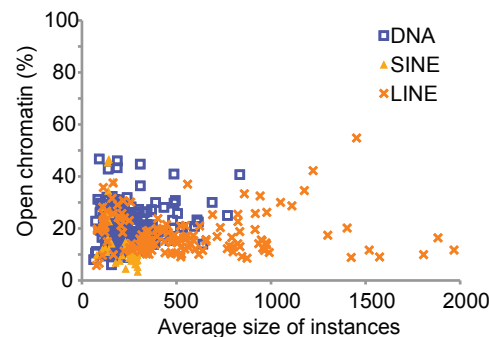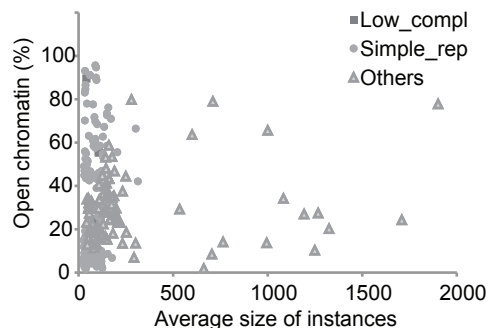**C**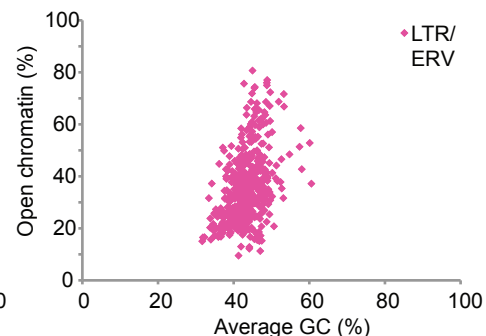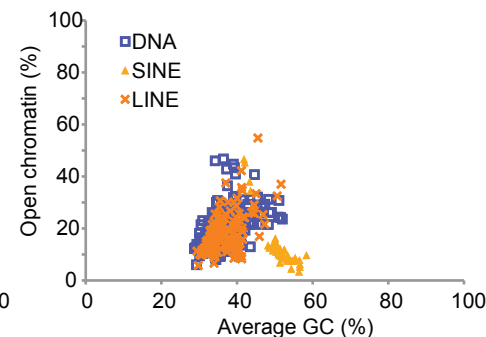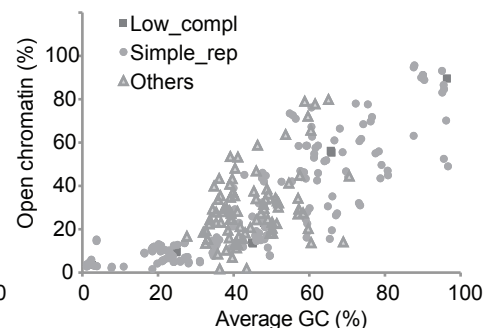

Supplement: Figure S2 — For each repeat subfamily, proportion of instances contributing to open chromatin in at least one data set (y-axis) relative to: (A) the number of instances, (B) their average size and (C) their average GC content. The only strong correlation is observed between the proportion of instances of Low_complex, Simple_rep and Others repeat subfamilies in open chromatin and GC content. (PDF) [file pgen.1003504.s002.pdf]

**A**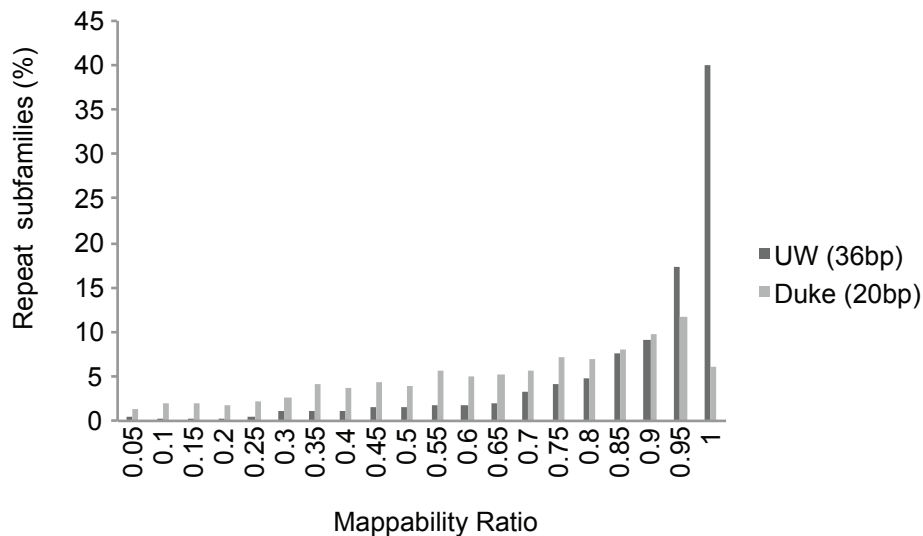**B**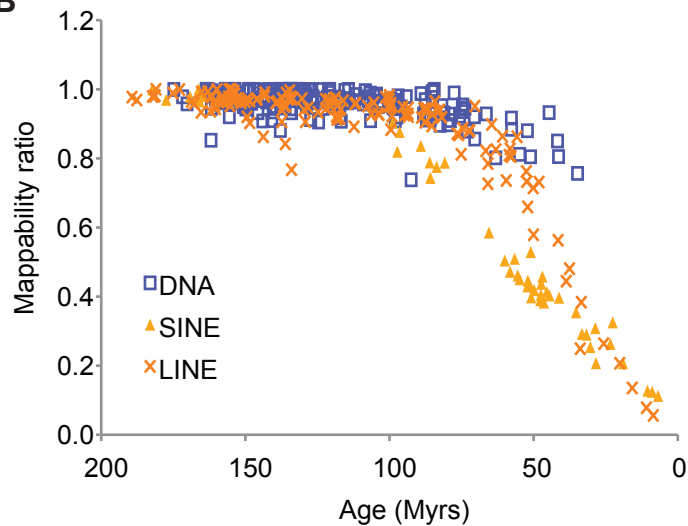**C**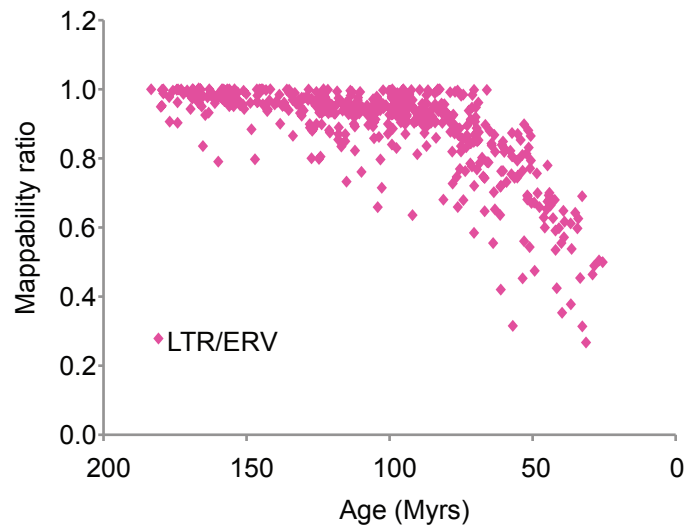

Supplement: Figure S3 — (A) Coverage of uniquely mapped short reads on repetitive regions. Fifty million random locations were selected from the human genome. For each of these locations 20 bp and 36 bp sequences were extracted to mimic UW and Duke DHS data sets. These artificial reads were re-mapped using Bowtie allowing for 1 mismatch and 2 mismatches respectively. For each repeat subfamily, a mappability ratio was computed as the number of reads uniquely mapped to this family divided by the number of artificial reads coming from this family. Overall we found that 36 bp reads perform significantly better than the 20 bp reads and 75% of the repeat families have a mappability ratio above 0.8. (B–C) Proportion of simulated reads that can be unambiguously mapped to the reference genome for all repeat subfamilies organized by class. Estimated age in millions of years (Myrs). (PDF) [file pgen.1003504.s003.pdf]

**A**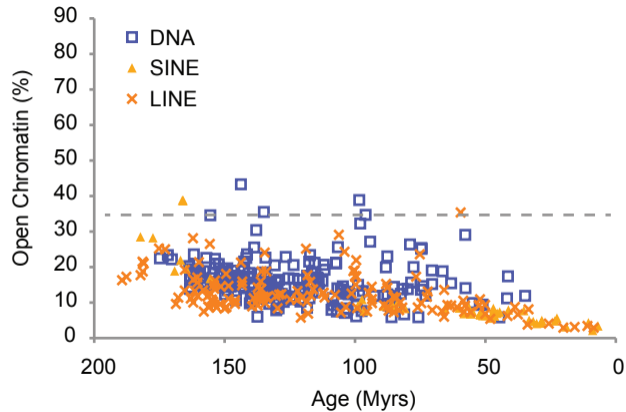**B**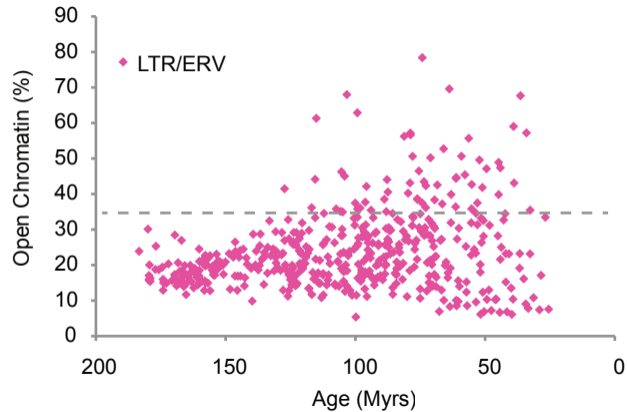

Supplement: Figure S4 — (A) Fraction of repeat instances in each DNA, SINE and LINE subfamily that is contributing to open chromatin in at least one normal data set. (B) Same for repeat subfamilies from the LTR/ERV class. In contrast to Figure 1E–1F this analysis is restricted to the normal cell lines. (PDF) [file pgen.1003504.s004.pdf]

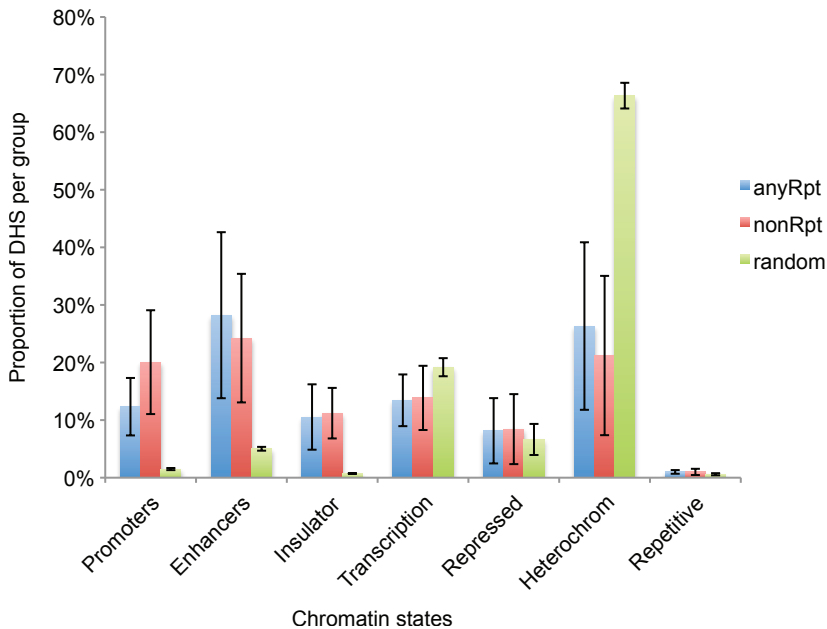

Supplement: Figure S5 — DHS overlapping repeats are enriched in active chromatin states at a similar level than the DHS outside repeats, and are enriched compared to a random distribution of the DHS. The 15 original states were combined into seven distinct states. The averages and standard deviations are calculated over the eight cell types for which DARs were identified, on the proportion of DHS overlapping (blue) or not (red) repeats or over random distribution (green). (PDF) [file pgen.1003504.s005.pdf]

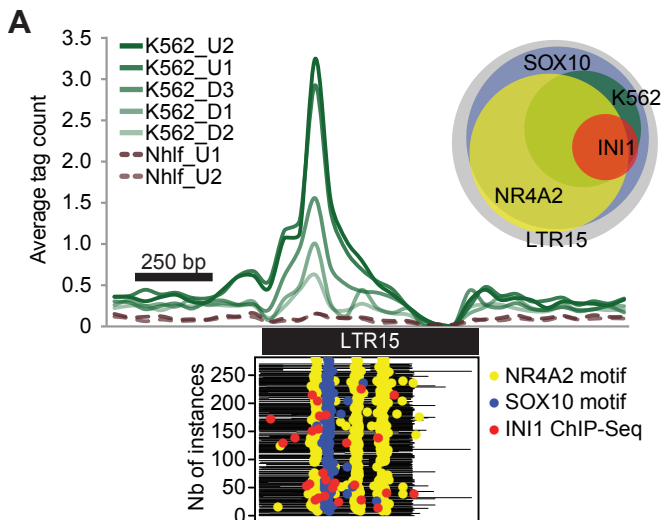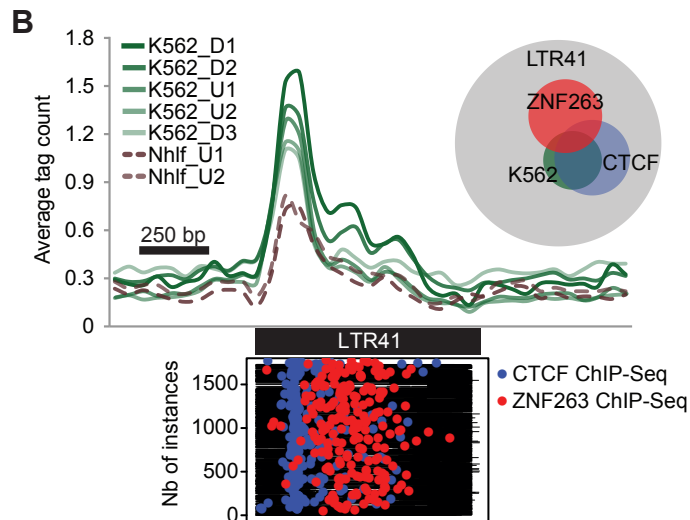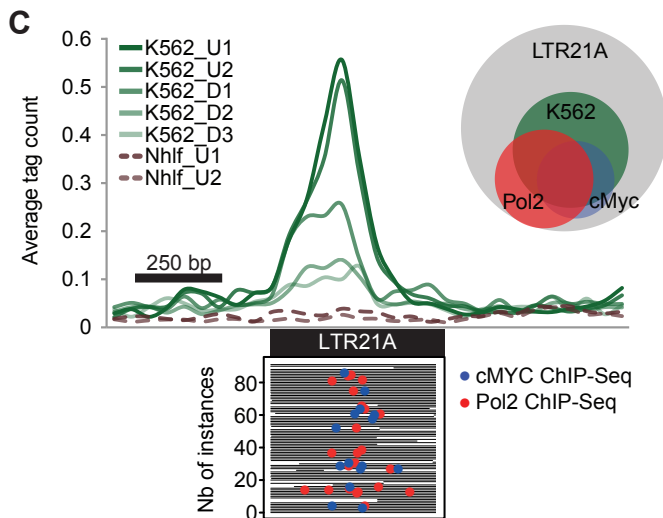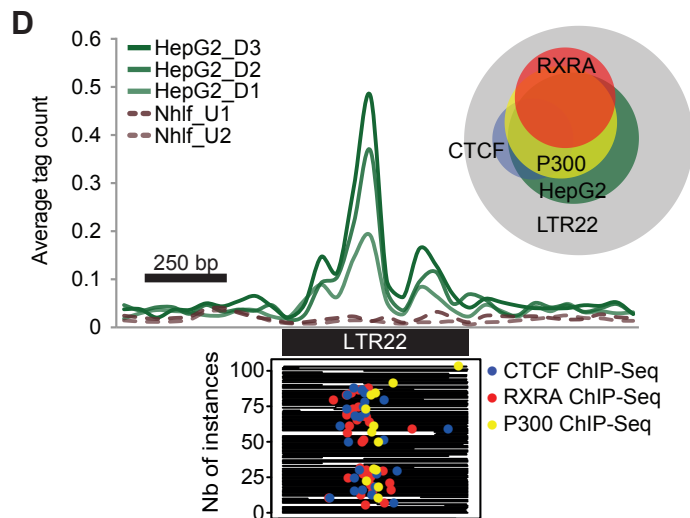

Supplement: Figure S6 — Complement of Figure 2 showing additional TF-repeat associations. Aggregate profiles of DNaseI tags (green) over the instances of different DARs: (A) LTR22 in HEPG2, (B) LTR15 in K562, (C) LTR41 in K562 and (D) LTR21B in K562. The profiles over another cell type (Nhlf) are shown as a control (dashed brown lines). The point's plots underneath the profiles represent the localization of regulatory motif or ChIP-Seq peaks in the same cell lines (yellow, blue, red points). The Venn diagrams represent the proportion of repeat instances (grey) containing DHS and regulatory motifs or ChIP-Seq peaks using the same color code. (PDF) [file pgen.1003504.s006.pdf]

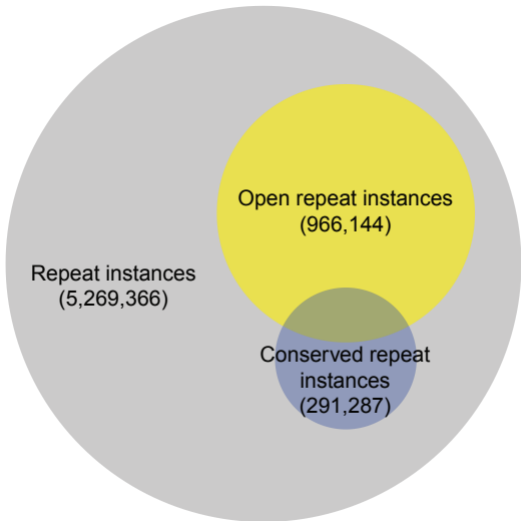

Supplement: Figure S7 — Repeat instances contributing to open chromatin tend to be more conserved than expected. Venn diagram showing the overall overlap of 87,219 between the Repeatmasker instances [43], the annotated conserved non-exonic elements (CNEEs) [29] and the DHS (this study). (PDF) [file pgen.1003504.s007.pdf]

**A**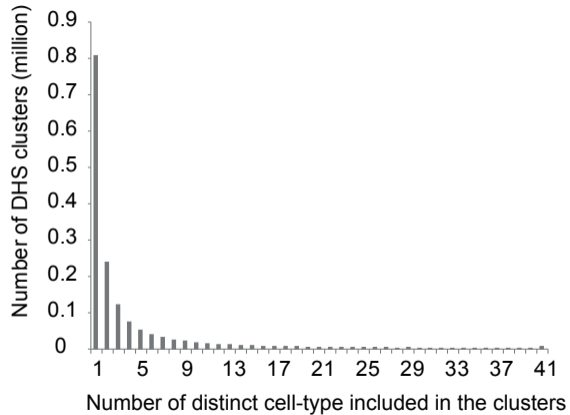**B**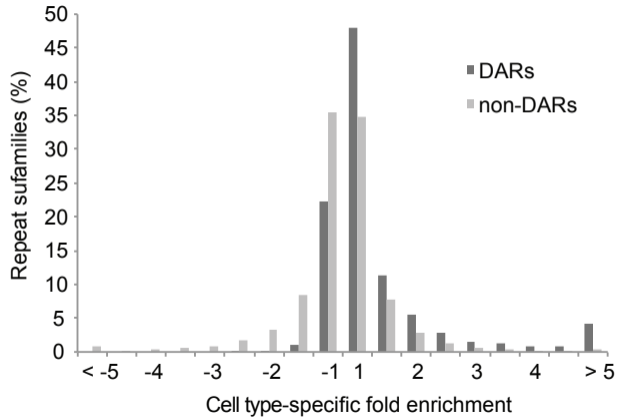

Supplement: Figure S8 — (A) Distribution of the number of cell types for all DHS regions showing that 75% of the clusters were contributing to open chromatin in 4 cell types or less. (B) Proportion of DARs and non-DARs repeat subfamily by bin of cell type-specific fold enrichment computed for each repeat subfamily in each data set. (PDF) [file pgen.1003504.s008.pdf]

**A**

LTR2B

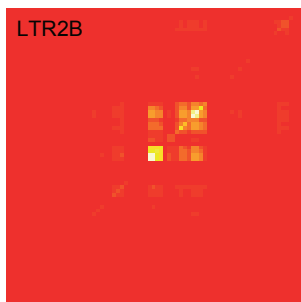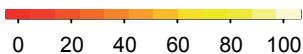**B**

LTR7

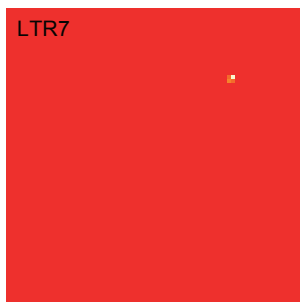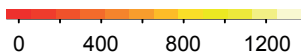**C**

MER121

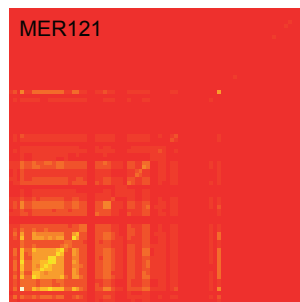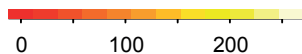**D**

LTR13

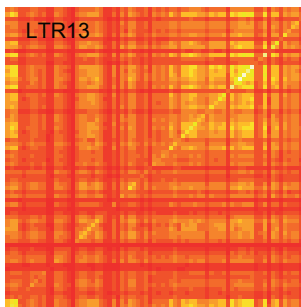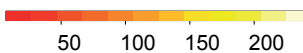**E**

LTR1

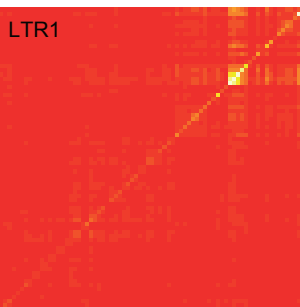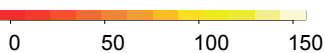**F**

LTR47B

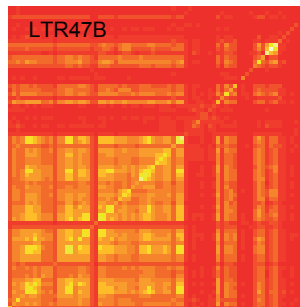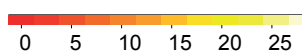**G**

LTR10C

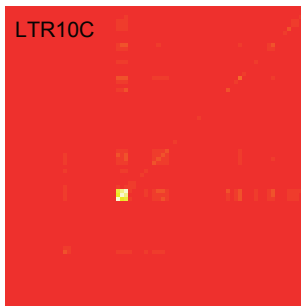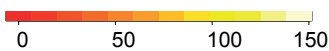**H**

LTR10A

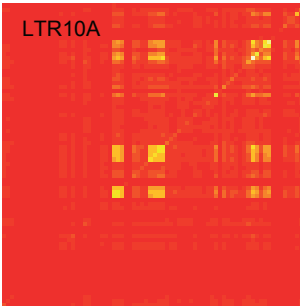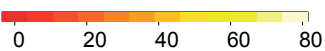**I**

LTR72

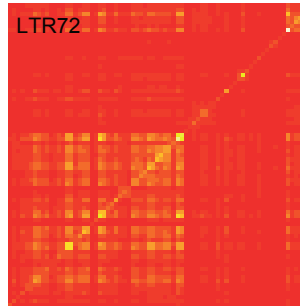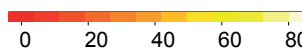

Supplement: Figure S9 — Cell type specific DAR examples. (A–C) LTR2B, LTR7 and MER121 from Figure 3. (D) LTR13 which is bound by CTCF (Figure 2B) and contributing to open chromatin in almost all cell type. (E) LTR1 which is showing re-activation of repeats in ESCs and some cancer cell lines but not in the others. (F) LTR47B which is contributing to open chromatin in many cell types except the lymphoblastoids, ESCs and leukemias. (G) LTR10C which is specifically contributing to open chromatin in HEE and SAEC epithelial cell types. (H) LTR10A which is contributing to open chromatin in few epithelial cell types as well as in solid tumors. (I) LTR72 which is contributing to open chromatin in many normal cell types and leukemia. (PDF) [file pgen.1003504.s009.pdf]

**A**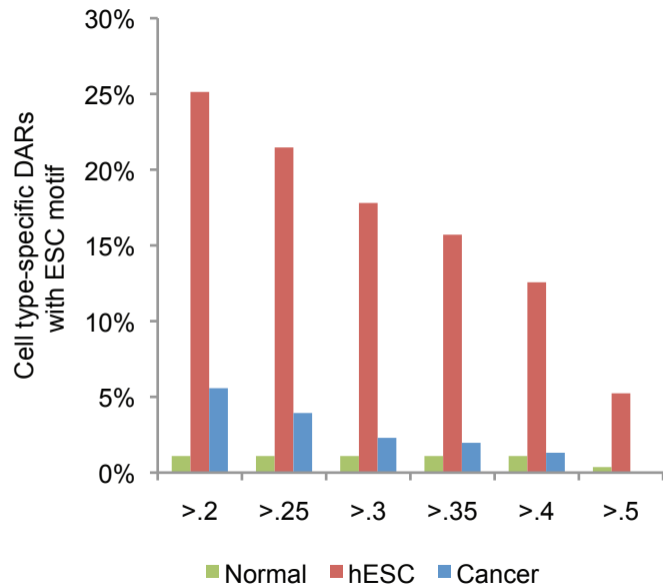**B**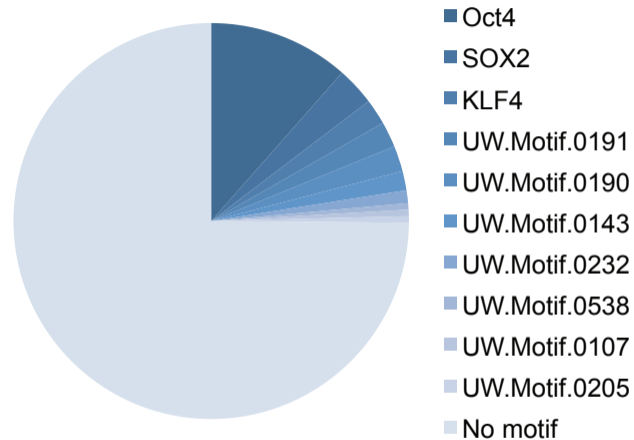

Supplement: Figure S11 — (A) Cell type-specific DARs with an ESC-specific motif as defined in [30] in different fraction of the DAR DHS instances (>.2, >.25, etc.) and with at least 25 motif instances. (B) ESC-specific motifs that are enriched in the ESC-specific DARs (with at least 25 motifs and >.2 of the DAR DHS instances). Only the most abundant motif per DAR is shown, but all combinations are available in Table S5. (PDF) [file pgen.1003504.s011.pdf]

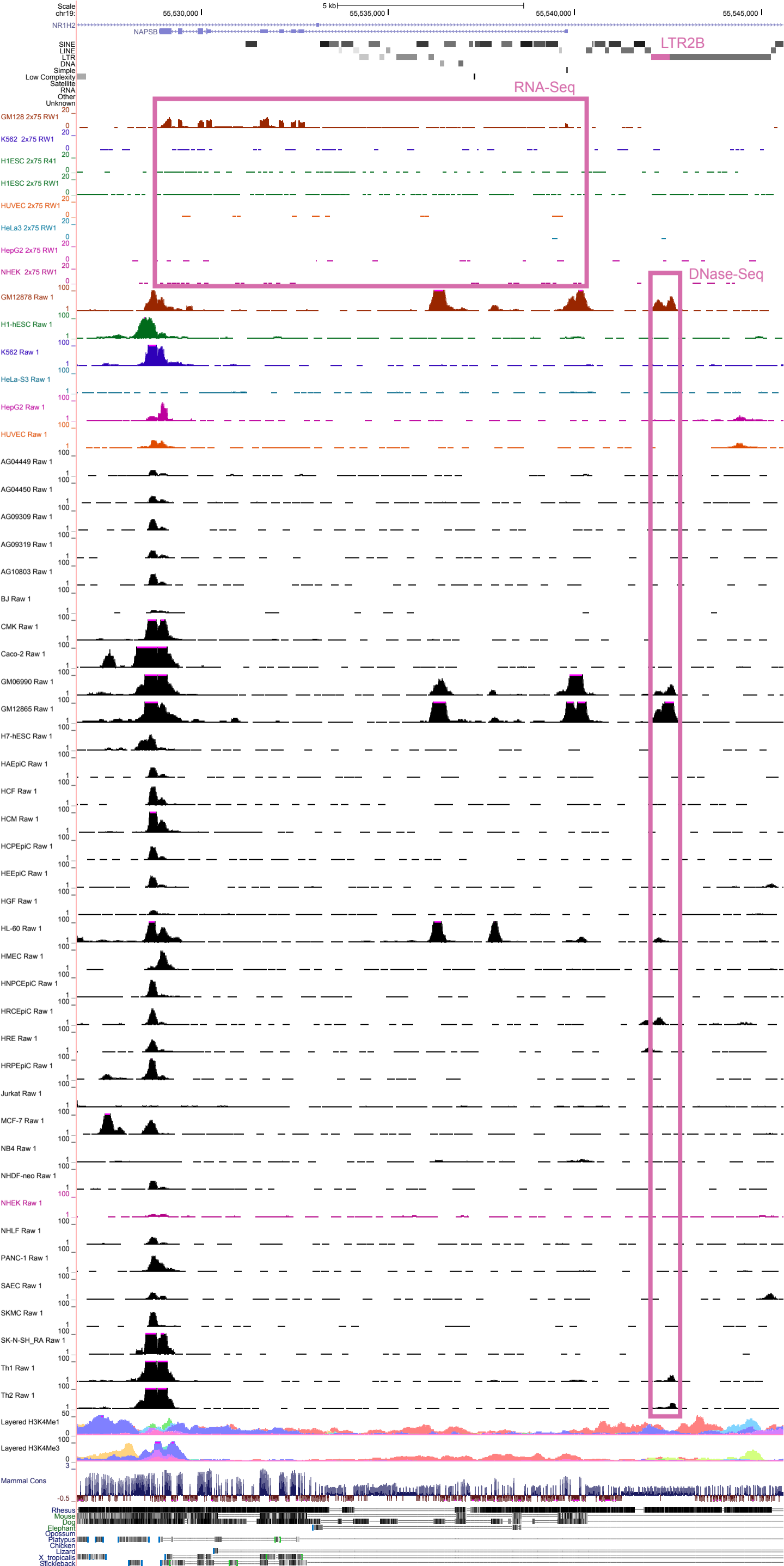

Supplement: Figure S12 — UCSC genome browser view of the NAPSB gene with RNA-Seq and DHS ENCODE tracks. The LTR2B repeat is highlighted in pink along with its cell type-specific contribution to open chromatin and expression profiles. (PDF) [file pgen.1003504.s012.pdf]

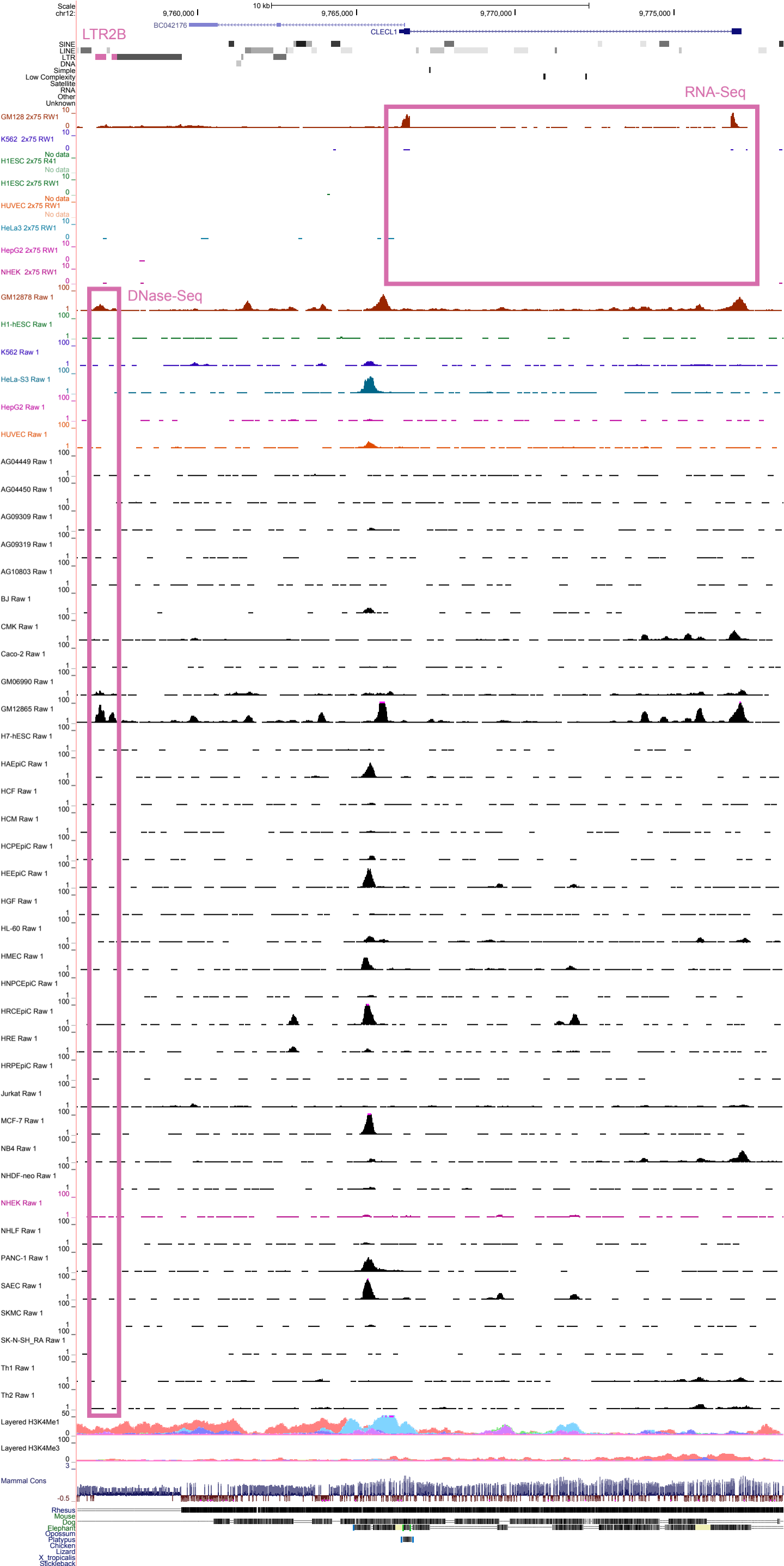

Supplement: Figure S13 — UCSC genome browser view of the CLECL1 gene with RNA-Seq and DHS ENCODE tracks. The LTR2B repeat is highlighted in pink along with its cell type-specific contribution to open chromatin and expression profiles. (PDF) [file pgen.1003504.s013.pdf]

**A**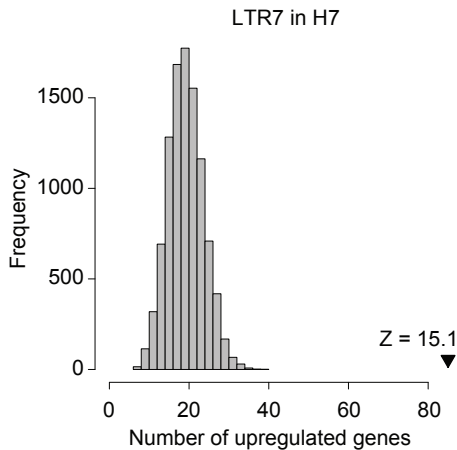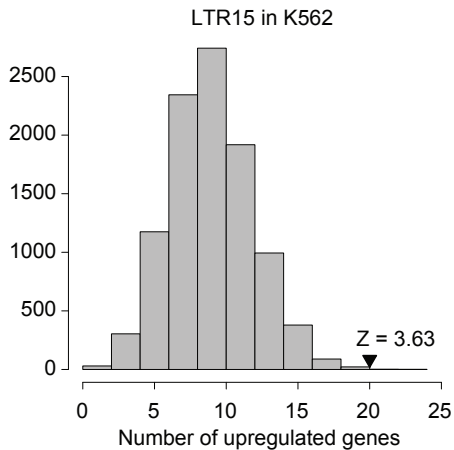**B**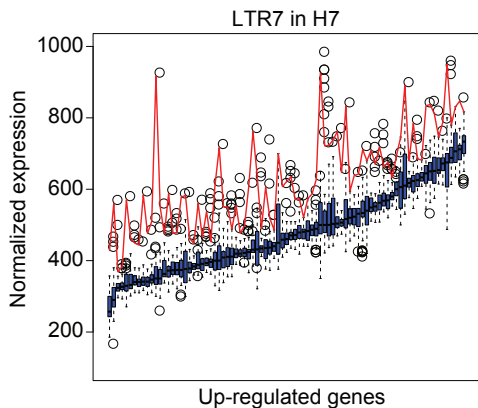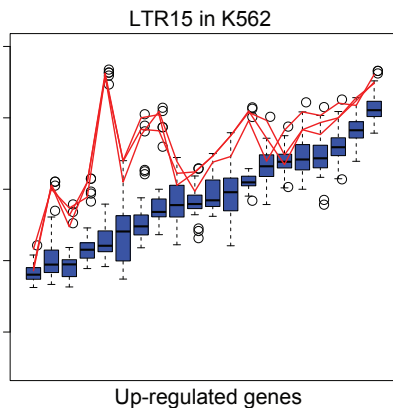

Supplement: Figure S14 — Cell type-specific expression of DAR-associated genes. (A) Distribution of the expected number of up-regulated genes in proximity to the DAR instances for LTR7 in H7 (left) and LTR15 in K562 (right). Actual number of up-regulated genes is shown using an arrowhead together with the corresponding Z-score. (B) Boxplots showing the expression values across cell types for the DAR-associated genes that are up-regulated. Red lines are connecting the expression values observed in the relevant cells. (PDF) [file pgen.1003504.s014.pdf]

**A**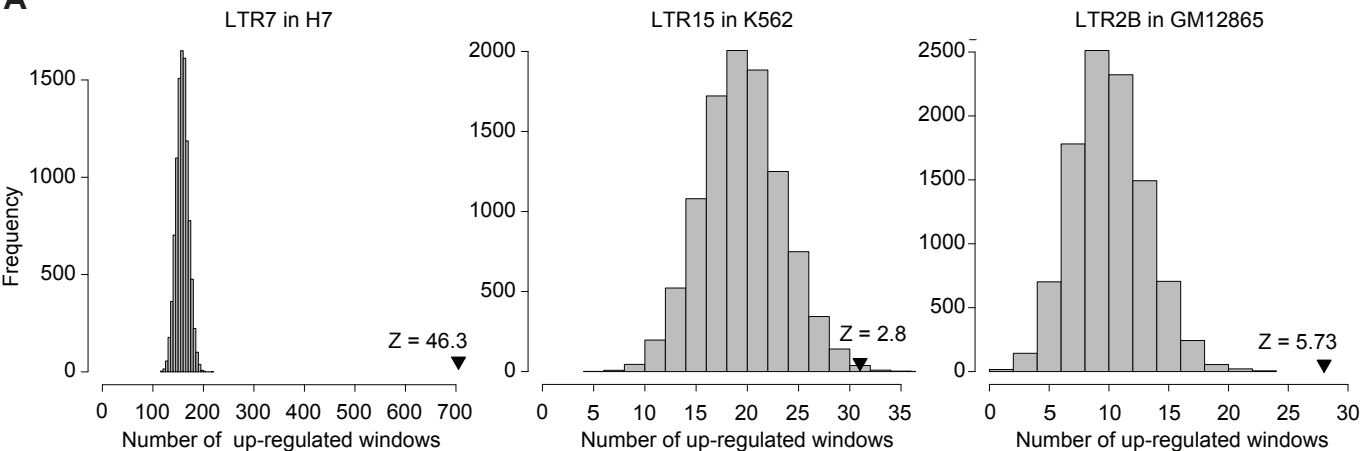**B**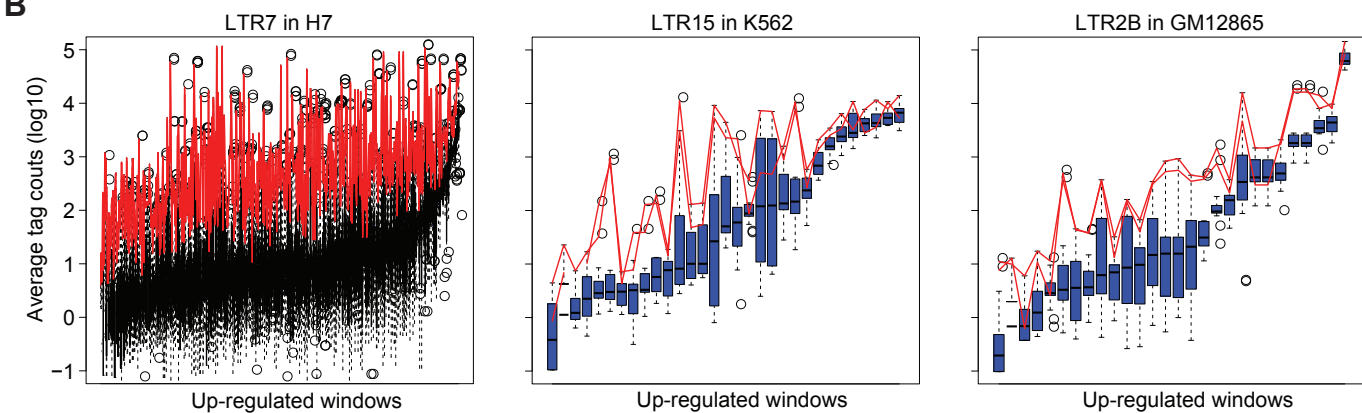**C**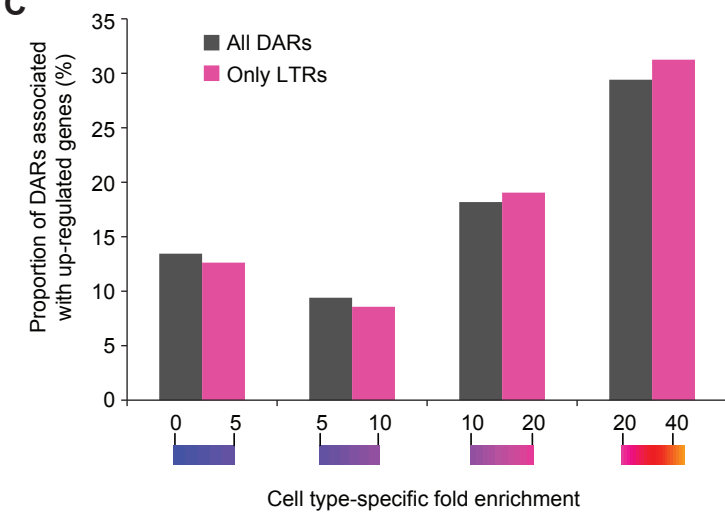

Supplement: Figure S15 — Cell type specific expression of the DARs based on RNA-Seq data. (A) Distribution of the expected number of up-regulated genes in proximity to the DAR instances for LTR7 in H7 (left), LTR15 in K562 (middle), and LTR2B in GM18265 (right). Actual number of up-regulated genes is shown using an arrowhead together with the corresponding Z-score. (B) Boxplots showing the expression values across cell types for the DAR-associated genes that are up-regulated. Red lines are connecting the expression values observed in the relevant cells. (C) Cell type-specific DARs tend to have more cell type-specific expression. DARs were binned according to their cell type-specific fold enrichment and the proportion of them having a Z-score of cell type-specificity expression above 3 is shown. (PDF) [file pgen.1003504.s015.pdf]
