# Supplementary material for: The Majority of Primate-Specific Regulatory Sequences Are Derived from Transposable Elements
Source: PLoS Genet. 2013 May 9;9(5):e1003504. doi: 10.1371/journal.pgen.1003504 (PMC3649963; doi:10.1371/journal.pgen.1003504)
Supplement: Figure S10 — Cell type-specific DARs are also supported by chromatin-state data. Repeat instances from specific DARs were intersected with the CS in the cell type where the DAR was identified (LTR7 in H1 and LTR2B in GM12878) but also in the other cell type. LTR13 was observed as a DAR in both H1 and GM12878. The CS result for LTR13 is consistent with the fact that the insulator protein CTCF was also enriched in this repeat subfamily (Figure 2B). (PDF) [file pgen.1003504.s010.pdf]

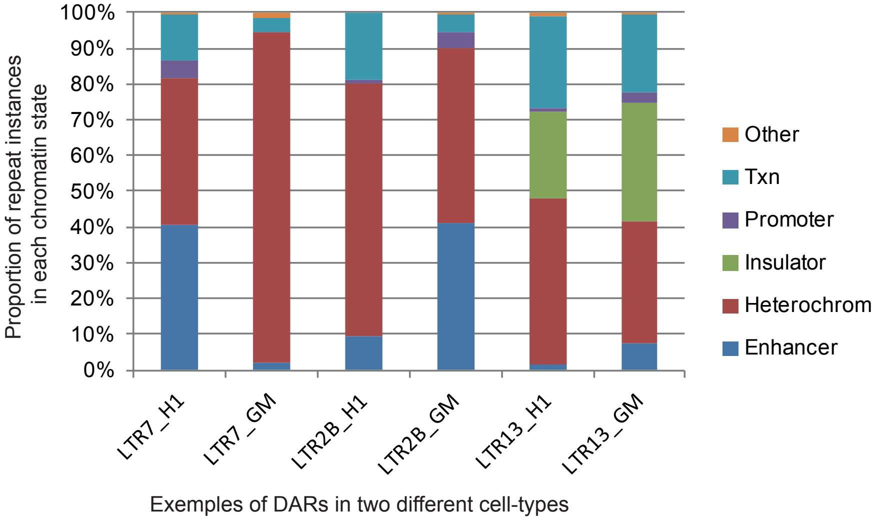
100%
90%
80%
70%
60%
50%
40%
30%
20%
10%
0%
Proportion of repeat instances
in each chromatin state
LTR7_H1
LTR7_GM
LTR2B_H1
LTR2B_GM
LTR13_H1
LTR13_GM
Exemples of DARs in two different cell-types
Other
Txn
Promoter
Insulator
Heterochrom
Enhancer
